# MitoInteractome: Mitochondrial protein interactome database, and its application in 'aging network' analysis

**DOI:** 10.1186/1471-2164-10-S3-S20

**Published:** 2009-12-03

**Authors:** Rohit Reja, AJ Venkatakrishnan, Jungwoo Lee, Byoung-Chul Kim, Jea-Woon Ryu, Sungsam Gong, Jong Bhak, Daeui Park

**Affiliations:** 1 Korean Bioinformation Center, KRIBB, Daejeon, 305-806, Korea; 2 Bioinformatics department, University of Science and Technology, Daejeon, 305-806, Korea; 3 Scotch College, 1 Morrison Street, Hawthorn, VIC 3122, Australia; 4 Department of Biochemistry, Biocomputing Group, University of Cambridge, Cambridge, CB2 1QW, UK

## Abstract

**Background:**

Mitochondria play a vital role in the energy production and apoptotic process of eukaryotic cells. Proteins in the mitochondria are encoded by nuclear and mitochondrial genes. Owing to a large increase in the number of identified mitochondrial protein sequences and completed mitochondrial genomes, it has become necessary to provide a web-based database of mitochondrial protein information.

**Results:**

We present 'MitoInteractome', a consolidated web-based portal containing a wealth of information on predicted protein-protein interactions, physico-chemical properties, polymorphism, and diseases related to the mitochondrial proteome. MitoInteractome contains 6,549 protein sequences which were extracted from the following databases: SwissProt, MitoP, MitoProteome, HPRD and Gene Ontology database. The first general mitochondrial interactome has been constructed based on the concept of 'homologous interaction' using PSIMAP (Protein Structural Interactome MAP) and PEIMAP (Protein Experimental Interactome MAP). Using the above mentioned methods, protein-protein interactions were predicted for 74 species. The mitochondrial protein interaction data of humans was used to construct a network for the aging process. Analysis of the 'aging network' gave us vital insights into the interactions among proteins that influence the aging process.

**Conclusion:**

MitoInteractome is a comprehensive database that would (1) aid in increasing our understanding of the molecular functions and interaction networks of mitochondrial proteins, (2) help in identifying new target proteins for experimental research using predicted protein-protein interaction information, and (3) help in identifying biomarkers for diagnosis and new molecular targets for drug development related to mitochondria. MitoInteractome is available at http://mitointeractome.kobic.kr/.

## Background

The mitochondrion, a membrane bound organelle, is often described as "the cellular power plant". It plays a critical role in energy production, metabolism, apoptosis, cellular proliferation, and other essential cellular processes. Consequently, mitochondrial dysfunctions result in disorders ranging from energy metabolism defects to neuro-degenerative diseases such as Parkinson's disease and Alzheimer's disease [1]. The association of mitochondria to these disorders has driven experimental approaches to define the mitochondrial proteome. Recently, proteomics approaches based on 2D-gel electrophoresis and mass spectrometry were employed to estimate the size of mitochondrial proteomes of individual species. Bardel *et al. *separated 433 protein spots in *Pisum sativum *(peas) mitochondrial proteome [2], while Kruft *et al. *separated 650 different *Arabidopsis thaliana *(thale cress) mitochondrial proteins [3] and estimated that 1,500 - 2,000 nuclear genes encode mitochondrial proteins. Taylor *et al. *separated 615 proteins from normal human heart tissue mitochondria and estimated the total human mitochondrial proteome to be about 1,500 proteins [4]. Owing to a steady increase in the amount of information, a comprehensive web-based information portal unifying mitochondrial proteome information has become a necessity.

In order to gain an in-depth understanding of the molecular functions and processes of mitochondria, it is essential to examine the interactions among its proteins. Here, we introduce 'MitoInteractome', a consolidated multi-species database for mitochondrial research that integrates information relevant to functions, pathways, diseases, non-synonymous SNP effects, protein-protein interactions, and physico-chemical properties. Further, we present an application of MitoInteractome in the construction and analysis of a protein interaction network for the 'aging' process.

## Construction and content

### Data source

The primary dataset for MitoInteractome was generated by performing a keyword search against SwissProt http://www.expasy.ch/sprot/ and including subsets from MitoP http://www.mitop.de:8080/mitop2/[5], MitoProteome http://www.mitoproteome.org/[6], HPRD http://www.hprd.org[7], and Gene Ontology (GO) database http://www.geneontology.org[8]. Proteins for which GO information was unavailable, in order to filter out false positives, we used the following algorithms that predict the sub-cellular localization of a given protein: 1) Psort http://psort.ims.u-tokyo.ac.jp/[9], 2) TargetP http://www.cbs.dtu.dk/services/TargetP/[10], and 3) Predotar http://urgi.versailles.inra.fr/predotar/predotar.html[11]. Only the proteins that were predicted to be mitochondrial by all the three algorithms were included. The refined primary dataset had 6,549 proteins spanning over 74 species. The flow-chart illustrating the process of collection of primary data and its refinement can be found in the supplement section of the MitoInteractome website http://mitointeractome.kobic.kr/supplement.php.

**Figure 1:**
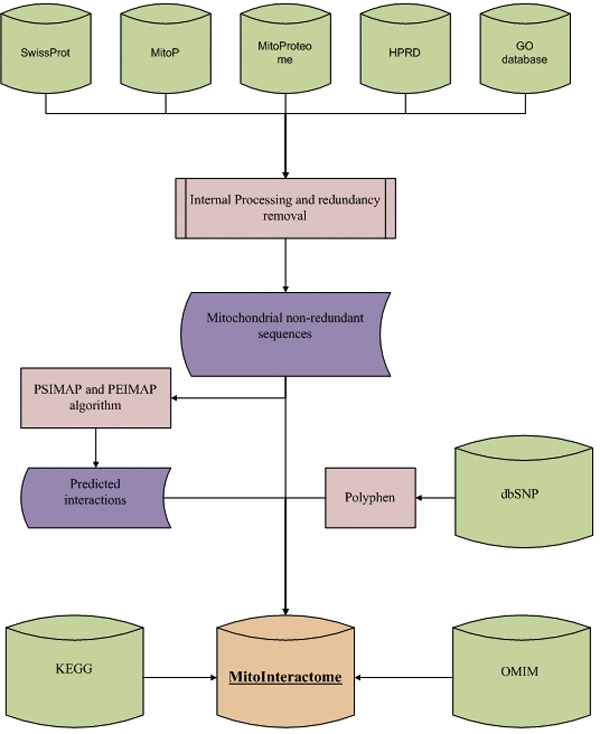
**Flow chart for the construction of MitoInteractome**. A schematic representation of the MitoInteractome database construction.

**Figure 2:**
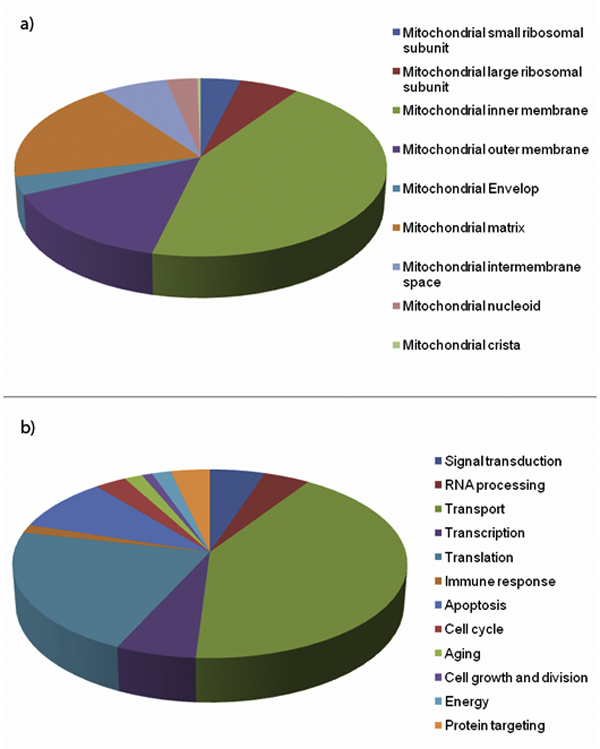
**Classification of proteins**. The classification of proteins based on (a) sub-mitochondrial location, and (b) functional process has been depicted using pie-charts.

Construction of complete MitoInteractome database is illustrated in Figure 1. We classified proteins based on their functional process and sub-mitochondrial localization when applicable. The classifications are illustrated in Figure 2.

### Protein interaction prediction

Protein-protein interactions are diverse, but all occur in a highly specific manner determined by the structural and physico-chemical properties of the interacting partners. We used two methods to predict protein interactions: PSIMAP (Protein Structural Interactome MAP) (http://psimap.com, http://psibase.kobic.re.kr) [12,13] and PEIMAP (Protein Experimental Interaction MAP) [14].

PSIMAP is a structure based interaction prediction method. It predicts interactions between known PDB http://www.rcsb.org/pdb domains based on the Euclidean distance between them. Two domains are assigned as 'interacting' if they have five or more atoms within 5 Ǻ distance. To construct the mitochondrial proteomes, we first assigned known 3D structural families in SCOP [15] to mitochondrial protein data sets. Among 6,549 mitochondrial proteins, 2,161 (32.99%) had at least one sequence homologous to the domain(s) in SCOP, based on the BLAST sequence similarity search with the following parameters: percentage identity: 40%, sequence coverage: 70%, and e-value: 10^-4^. However, the same region of a protein sequence could be assigned and overlapped by more than two SCOP families, each of which falls within the same significance level. In these cases, we accepted overlaps of up to 15 amino acid residues in the alignments and regarded them as two separate domains for a given protein sequence. Using PSIMAP, we found 36,359 structural interaction pairs from the mitochondrial proteomes.

Structural interaction information tends to provide more number of predictions as many specifically different sequences share the same structure. Therefore, experiment based sequence based PPI data are usually more specific. Our usual step is to predict many partners using structural interaction information and then apply sequence based experimental (more specific incident of protein-protein interaction) PPI data to filter out the first.

PEIMAP (Protein Experimental Interaction MAP) uses experimental data available in BIND http://bond.unleashedinformatics.com[16], DIP http://dip.doe-mbi.ucla.edu[17], MINT http://mint.bio.uniroma2.it/mint[18], IntAct http://www.ebi.ac.uk/intact[19], and HPRD interaction databases as a basis for prediction. The interactions are predicted based on the concept of 'homologous interaction', which means that if two proteins in a species, are known to be interacting, then their respective orthologs in another species are also likely to interact. An illustration of the concept of homologous interaction and the required parameters for the same are available in the supplement section of MitoInteractome's website. The mitochondrial proteins were aligned with the protein sequences involved in, experimentally known, protein interactions using BLAST (cut-off sequence identity of 80%). Using PEIMAP we found 23,973 interaction pairs.

### Disease related information, nsSNPs and their effects

Mitochondrial dysfunction is associated with a large number of aging-related neurodegenerative diseases such as Alzheimer's disease, Parkinson's disease, Amyotrophic lateral sclerosis, and Huntington's disease [20]. To date, 102 heritable disorders have been attributed to defects in a quarter of the known nuclear-encoded mitochondrial proteins in humans. More than 60 disorders have been reported to be derived from mutations in the human mitochondrial genome. Many mitochondrial diseases remain unexplained, however, partly because only 40~60% of the presumed 700-1,000 proteins involved in mitochondrial function and biogenesis have been identified [21]. Our primary source of information on diseases was OMIM http://www.ncbi.nlm.nih.gov/Omim/[22]. The number of base substitutions sites in the mitochondrial genome related to cytopathies was 74 [23]. Information associated with mitochondrial diseases can be accessed using the search form available at http://mitointeractome.kobic.kr/search.php.

It is widely accepted that polymorphisms in the human genome are expected to play a key role in defining the etiologic basis of phenotypic differences amongst individuals in the aspect of drug responses and common disease predisposition [24]. Non-synonymous SNPs are known to be closely associated with inherited diseases. MitoInteractome contains 1301 non-synonymous SNPs, collected from dbSNP http://www.ncbi.nlm.nih.gov/projects/SNP/ build 129 [25], associated with 512 human mitochondrial proteins. We analysed the non-synonymous SNPs using PolyPhen http://genetics.bwh.harvard.edu/pph/[26], a tool that predicts the impact of non-synonymous SNPs on structure and function of proteins. The non-synonymous SNPs were classified based on the confidence score of their potency to be deleterious. 321 substitutions were predicted to be probably damaging and 226 were predicted as possibly damaging, while the rest were predicted as benign substitutions.

### Pathway information

Pathway information is vital for successful quantitative modeling of biological systems. Mitochondria have been implicated in the origin of diseases, because of their crucial role in energy production, in nuclear cytoplasmic signal integration, and in the control of metabolic pathways. Mitochondrial pathway information comprising of pathways, enzymes involved, and their respective KEGG Orthology was collected from KEGG http://www.genome.jp/kegg/[27]. The pathway information can be used to perform flux balance analyses to estimate the metabolic capabilities of the mitochondria. Enzymes were referenced to BRENDA http://www.brenda-enzymes.info/[28].

## Utility and discussion

### Web interface

MitoInteractome is accessible through a web interface that is powered by PHP and JavaScript. The data is stored in retrievable MySQL tables.

**Figure 3:**
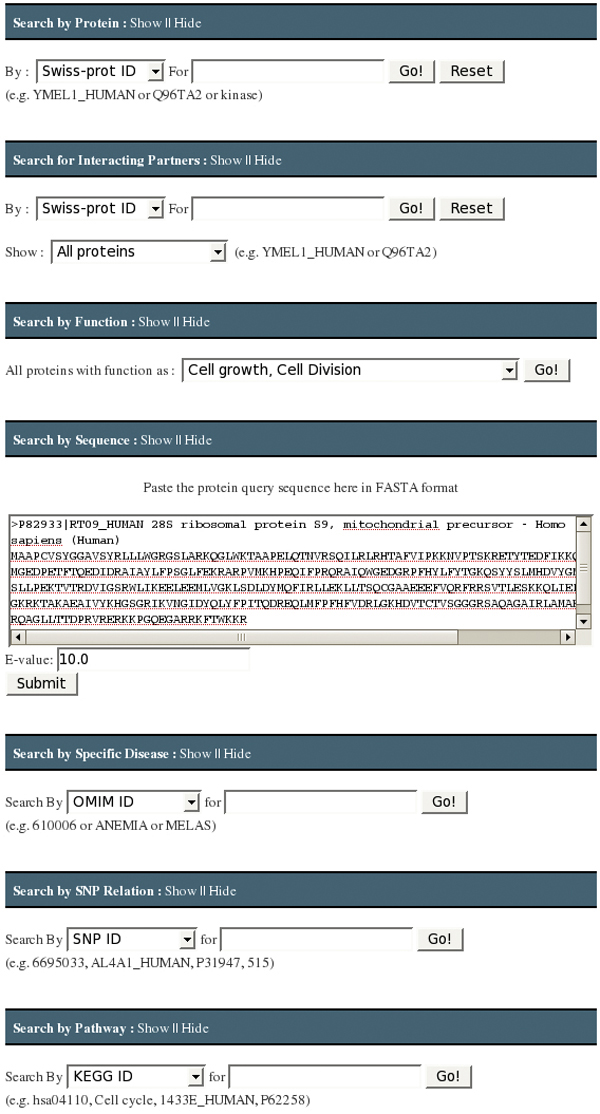
**The graphical user interface for MitoInteractome**. The graphical user interface for MitoInteractome providing facility to search by 1) Protein 2) Interacting partners 3) Function 4) Sequence 5) Disease association 6) SNP relation 7) Pathway association.

**Figure 4:**
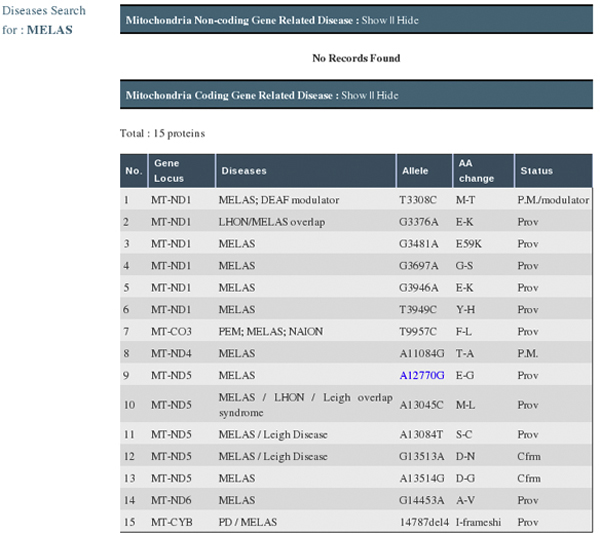
**Result page showing disease information with associated variation**. The result set obtained by using the interface *Search by Disease*, with search criteria: disease name *e.g. *"MELAS". The result displays the disease information with associated variation.

As shown in Figure 3, the MitoInteractome can be queried, using primary identification number/accession number (from SwissProt), protein name/non-synonymous SNP positions, and disease names. The facility to limit the interacting partners to human proteins is available. The sequences stored in the database can be searched for high similarity matches using BLAST, which is built into the website. The summary of search results shows the following: interacting partners, SNPs within proteins, the number of SNPs, disease related proteins, gravy scores, physicochemical properties, pathway information, and cross-references to external databases. For a search performed using a disease name, MitoInteractome provides detailed disease information with associated variations (Figure 4).

**Figure 5:**
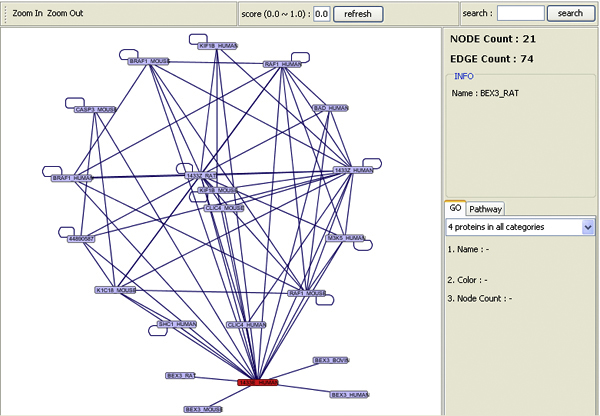
**Java tool for interaction visualization**. The interaction map for protein *e.g*. [SwissProt: 1433E_HUMAN].

We developed a Java based network viewer to graphically visualize protein-protein interactions (Figure 5). Information on the construction of MitoInteractome and algorithms used are available on the supplement page of the website. Interaction information is available for download in tab-deliminated format, Cytoscape SIF format, and PSI MI 2.5 format from the download page http://mitointeractome.kobic.kr/downloads.php. The help page explains the function of every search interface.

### 'Aging network' - construction and analysis

The process of 'aging' is one of the active fields of biological research, the study of the biological process aspect of which is popular as 'Biogerentology'. It is common knowledge that the onset of several neurodegenerative disorders such as Alzheimer's disease, Parkinson's disease, Leigh's syndrome etc. are of progressive nature with aging. Several studies in the past have indicated association of the aging process with mitochondria [29-31]. Since functioning of the mitochondria is dependent on the number of its constituent components, it is highly likely that a complex network of interacting proteins is involved in the aging process. Although previous studies have pointed towards individual proteins associated with the aging process, interactions among them have not been investigated.

**Figure 6:**
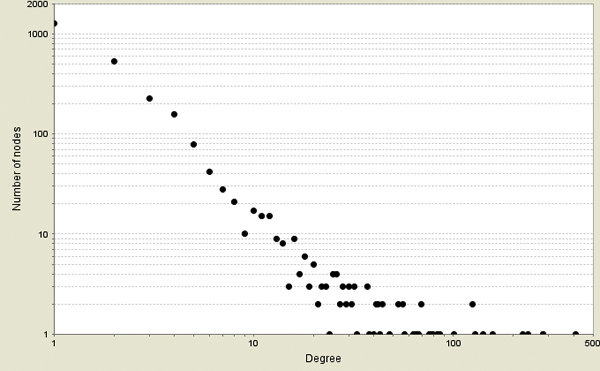
**Plot of node degree distribution**. The figure illustrates the node degree distribution for the aging network constructed.

**Figure 7:**
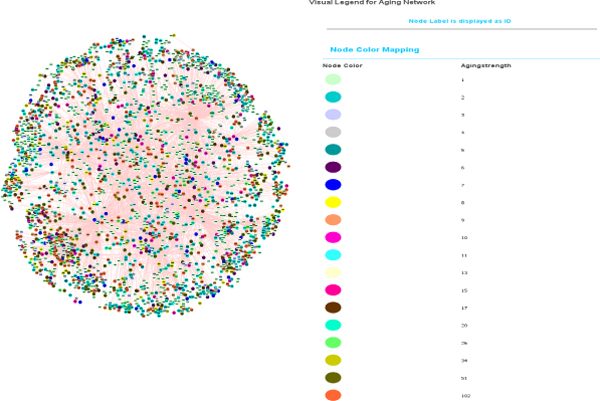
**'Aging network' visualized using Cytoscape**. The graphical view of the 'aging network' where proteins are denoted using nodes and interactions are denoted using edges. The nodes are colored based on the strength centrality measure. Cytoscape http://www.cytoscape.org/ was used for network visualization.

A list of 42 distinct proteins associated with the 'aging' process was obtained from the GO database [32]. These proteins were queried against MitoInteractome to find the probable interacting partners of the proteins associated with aging. The network constructed had 2,527 nodes (proteins) and 5,356 edges (interactions). The plot of the node degree distribution is shown in Figure 6. Nodes were assigned weights based on the uniqueness of its representative protein across 102 eukaryotes that were collected from KEGG. Every protein was decomposed into its constituent PFAM [33] domains and the conservation of the combination of domains was scored on a scale of 0 - 1. The proteins that had the least conserved domains, and hence were unique to this network, were weighted as 1, while proteins that were most conserved were weighted as 0. The network is illustrated in Figure 7.

A centrality measure - "Strength of a node" - was introduced, which is defined as the sum of the weights of other nodes it is interacting with. Strength for a weighted network is analogous to 'Connectivity' for an un-weighted network. Strength of a node in the present network would capture the size of its neighborhood, constituted by the interacting proteins, as well as the uniqueness of the neighborhood, derived from the domain conservation analysis. Proteins possessing higher strengths (*e.g *Gene name: *GRB2, ABL1, TP53, ANDE1*) were observed to be involved in at least one aging related disease, while proteins (*e.g. *Gene name: *ACTB, HNRNPA3, EIF4H*) that had low strengths were observed to be mostly house-keeping gene products.

*GRB2 *is known to be involved in embryonic development and malignant transformation [34]. Due to its involvement as a signal transducer in pathways, including pathways and genes previously related to aging such as *INS/IGF1 *signalling and *SHC1 *[35], it is probable that *GRB2 *plays a vital role in ageing. *GRB2 *was observed to have the highest strength of 75.76.

*ABL1 *plays an important role in cellular proliferation and development [36]. They are activated by oxidative stress and are related to aging and tumorigenesis. It was found that disruption of *ABL1 *in mice results in several defects and leads to death shortly after birth [37]. It is present among the central proteins of the aging network with a strength of 46.16.

It has previously been reported that the p53 protein (encoded by *TP53 *gene) plays an important role in aging. Matheu *et. al. *[38] showed that genetically modified mice with increased levels of p53 have decreased levels of aging associated damage. In the aging network constructed the p53 protein was observed to have a high strength of 45.97. The *TP53 *gene acts as a tumor suppressor and also induces apoptosis depending on the physiological circumstances.

*E1 *is ATP dependent DNA helicase required for initiation of viral DNA replication and are involved in almost all aspect of nucleic acid metabolism including replication, repair, recombination, transcription, translation and ribosome biogenesis. Defects in helicase gene have been reported to be responsible for variety of human genetic disorders which can lead to cancer, premature aging or mental disorders. It can be inferred that *E1*, which has a strength of 42.05, might be significant to the aging network [39].

Other strong proteins and their associated functions are listed in Table 1. From the observations made in the 'aging network' analysis, it is reasonable to conjecture that proteins having high strengths in the analyzed network are the key players in aging network and could influence the longevity of the organism.

**Table 1:** List of the proteins with high strength scores in the human 'aging network'.

Genes	Common Names	Function	Score
*GRB2*	Growth factor receptor-bound protein 2	SH3/SH2 adaptor activity	75.76
*NCK1*	Cytoplasmic protein NCK1	Receptor binding	61.24
*ABL1*	Proto-oncogene tyrosine-protein kinase ABL1	ATP binding	46.16
*TP53*	Cellular tumour antigen p53	Apoptosis	45.97
*E1*	Replication protein E1	ATP binding	42.05
*YWHAZ*	14-3-3 protein zeta/delta	Anti-apoptosis	37.83
*KIAA1377*	Uncharacterized protein KIAA1377	Protein binding	36.87
*EEF1A1*	Elongation factor 1-alpha 1	Protein binding	36.45
*UNC119*	Protein unc-119 homolog A	Synaptic transmission	33.69
*HSPA8*	Heat shock cognate 71 kDa protein	ATP binding	33.08

## Conclusion

MitoInteractome is a comprehensive database containing the following information on proteins in mitochondria: physico-chemical properties, predicted interaction partners, polymorphism and their effects, diseases and pathways involved. The database has been cross referenced to external database where necessary. The derived information stored in MitoInteractome would serve as a precursor for the construction and analysis of protein networks.

The construction and analysis of 'aging network' presented in this work as a case-study provided us with insights into interactions among the key entities involved in the aging process. The same protocol can be extended to construct similar interaction networks and investigate other mitochondria centric processes such as Apoptosis, Oxidative Phosphorylation.

## Availability and requirements

MitoInteractome can be accessed at http://mitointeractome.kobic.kr/. Java version 1.5 or later will be required to view the predicted interactions.

## List of abbreviations used

BIND: Bio molecular Interaction Network Database; DIP: Database of Interacting Proteins; HPRD: Human Protein Reference Database; KEGG: Kyoto Encyclopaedia of Genes and Genomes; MINT: Molecular INTeraction database; OMIM: Online Mendelian Inheritance in Man; PDB: Protein Data Bank; PEIMAP: Protein Experimental Interactome MAP; PSIMAP: Protein Structural Interactome MAP; SCOP: Structural Classification of Proteins; SNP: Single Nucleotide Polymorphism.

## Competing interests

The authors declare that they have no competing interests.

## Authors' contributions

VAJ and RR did most of the work. VAJ collected the dataset and RR did the website designing and the aging analysis. JL and SG made disease mapping pipeline and PEIMAP pipeline initially and helped in manuscript writing. BCK and JWR provided us with the PPI prediction information. DP did system design and provided useful discussion. JB had the original idea and study.

## Note

Other papers from the meeting have been published as part of *BMC Bioinformatics* Volume 10 Supplement 15, 2009: Eighth International Conference on Bioinformatics (InCoB2009): Bioinformatics, available online at http://www.biomedcentral.com/1471-2105/10?issue=S15.
